# Serum Linkage-Specific Sialylation Changes Are Potential Biomarkers for Monitoring and Predicting the Recurrence of Papillary Thyroid Cancer Following Thyroidectomy

**DOI:** 10.3389/fendo.2022.858325

**Published:** 2022-04-29

**Authors:** Zhen Cao, Zejian Zhang, Rui Liu, Mengwei Wu, Zepeng Li, Xiequn Xu, Ziwen Liu

**Affiliations:** ^1^ Department of General Surgery, Peking Union Medical College Hospital, Chinese Academy of Medical Sciences and Peking Union Medical College, Beijing, China; ^2^ Department of Medical Research Center, State Key Laboratory of Complex Severe and Rare Diseases, Peking Union Medical College Hospital, Chinese Academy of Medical Sciences and Peking Union Medical College, Beijing, China; ^3^ Department of Clinical Laboratory, Peking Union Medical College Hospital, Chinese Academy of Medical Sciences and Peking Union Medical College, Beijing, China

**Keywords:** papillary thyroid carcinoma, recurrence, biomarker, *N*-glycosylation, serum glycomics

## Abstract

**Background:**

Although papillary thyroid cancer (PTC) could remain indolent, the recurrence rates after thyroidectomy are approximately 20%. There are currently no accurate serum biomarkers that can monitor and predict recurrence of PTC after thyroidectomy. This study aimed to explore novel serum biomarkers that are relevant to the monitoring and prediction of recurrence in PTC using *N*-glycomics.

**Methods:**

A high-throughput quantitative strategy based on matrix-assisted laser desorption/ionization time-of-flight mass spectrometry was used to obtain serum protein *N*-glycomes of well-differentiated PTC, postoperative surveillance (PS), postoperative recurrence (PR), and matched healthy controls (HC) including linkage-specific sialylation information.

**Results:**

Serum *N*-glycan traits were found to differ among PTC, PS, PR, and HC. The differentially expressed *N*-glycan traits consisting of sixteen directly detected glycan traits and seven derived glycan traits indicated the response to surgical resection therapy and the potential for monitoring the PTC. Two glycan traits representing the levels of linkage-specific sialylation (H4N3F1L1 and H4N6F1E1) which were down-regulated in PS and up-regulated in PR showed high potential as biomarkers for predicting the recurrence after thyroidectomy.

**Conclusions:**

To the best of our knowledge, this study provides comprehensive evaluations of the serum *N*-glycomic changes in patients with PS or PR for the first time. Several candidate serum *N*-glycan biomarkers including the linkage-specific sialylation have been determined, some of which have potential in the prediction of recurrence in PTC, and others of which can help to explore and monitor the response to initial surgical resection therapy. The findings enhanced the comprehension of PTC.

## Introduction

Papillary thyroid carcinoma (PTC) is one of the fastest-growing malignancies and has a relatively excellent long-term prognosis. The standard treatment strategies of PTC include surgical resection, radioactive iodine (RAI) ablation, and thyroid-stimulating hormone (TSH) suppressive therapy, causing an excellent 5-year overall survival (OS) of 98.2% (1, 2). Although PTC is an indolent tumor, it frequently spreads to the regional and distant lymph nodes. Even with modern surgical and medical management, the rate of recurrence after initial surgery was estimated to be about 20% in contrast to the good survival outcomes. Subsequent recurrence of PTC after thyroidectomy may lead to severe surgical trauma (3). Therefore, it is crucial for early identification of which patients are at high risk of recurrence (4–6). The ability to predict recurrence not only could provide patient counseling, but also help surgeons guide management decisions. The routine examination procedures for recurrence during the follow-up period generally rely on ultrasound, ultrasound-guided percutaneous fine-needle aspiration (FNA) cytology, and serum thyroglobulin (Tg) measurement (7). However, these approaches suffer from apparent shortcomings. The sensitivity of lymph node metastasis (LNM) detection by ultrasound examination is low at about 41.3-61% (8). FNA cytology is chosen to confirm the nature of suspicious lymph nodes but cytological uncertainty is present in 20% to 30% of FNA samples. False-negative Tg rates ranging from 4% to 35% in thyroid cancer patients with evidence of regional and distant metastatic disease had been reported in multiple studies (9–11). To sum up, it is urgent to identify novel and non-invasion biomarkers that could be complemented to ultrasound, FNA, and serum Tg level to improve the accuracy of predicting recurrence for PTC patients after thyroidectomy.

Glycosylation is one of the most common and important post-translational modifications of proteins which can greatly affect the structure and function of proteins and its influence in biological processes such as protein degradation, secretion, transport, and immune response (12–14). Protein glycomic characteristics could dramatically vary due to the pathologic conditions and it has been found that aberrant glycosylation can be the result of initial carcinogenic transformation (15, 16). These dynamic changes indicated that aberrant glycosylation can serve as biomarkers or treatment targets (17). Moreover, multiple prior studies revealed that altered glycosylation is related to tumor initiation, invasion, and metastasis. The interrogation of glycosylation profiles in the broader context of cancer can provide important insight into the underlying mechanisms regarding cancer progression or recurrence. In current years, serological glycomics profiling provides a new method for the exploration of non-invasive biomarkers. Approaches based on glycomics have been widely used for the identification of biomarkers in thyroid cancer (18–22). These studies mainly focused on searching diagnosis biomarkers or therapeutic targets (20, 23). However, the potential of glycans as monitor or prognosis biomarkers has not previously been investigated.

In the present study, serum *N*-glycomic profiles of four subgroups including PTC, PS, HC, and PR were evaluated. Since the functions of sialylation depend on the linkage type, this workflow applied linkage-specific sialic acid derivatization to distinguish α-2,3- and α-2,6-linked sialic acids on the serum released *N*-glycans. The detailed analysis using a high-throughput quantitative strategy based on matrix-assisted laser desorption/ionization time-of-flight mass spectrometry (MALDI-TOF MS) and automated data processing were performed (24, 25). We sought to compare and reveal the differences in the serum *N*-glycome of PTC, PS, HC, and PR and identify novel serum glycan biomarkers for monitoring and predicting recurrence of PTC following thyroidectomy, as well as provide insight in exploring the response to initial surgical resection therapy.

## Materials and Methods

### Study Population and Serum Sample Collection

Serum specimens obtained from 80 patients diagnosed with PTC, 86 PTC patients undergoing initial thyroidectomy during the PS, 80 HC volunteers, and 9 postoperative recurrence PTC patients were consecutively recruited in Peking Union Medical College Hospital (Beijing, China) between June 2019 to November 2020. The four subgroups were sex- and age-matched as far as possible. Patients with PTC were diagnosed based on thyroid ultrasonography and FNA, confirmed by pathologists, and had no distant metastasis. The inclusion criteria for PS patients were: (a) patients with no postoperative clinical evidence of lymph node involvement by ultrasonography; (b) patients who underwent total thyroidectomy with prophylactic central neck dissection with at least 10 sampled nodes; (c) the low serum Tg and TSH levels; (d) with a minimum follow-up period of 12 months. The inclusion criteria for PR patients were: (a) patients who underwent total thyroidectomy with prophylactic central neck dissection with at least 10 sampled nodes; (b) suspicious recurrent lymph nodes were identified by imaging, FNA, or core-needle biopsy; (c) follow-up at regular intervals of thyroid ultrasonography and thyroid function. The following exclusion criteria were applied in the PTC, PS, and PR group: (a) with history of systemic, autoimmune, infectious, or blood diseases; (b) with impaired glucose tolerance or diabetes; (c) history of pituitary disease or other malignancies. All volunteers of the HC group were defined by general practitioners. They should have normal thyroid function, normal thyroid ultrasonography, normal biochemical parameters, normal blood coagulation parameters, and no history of systematic diseases. Ultrasound was performed by the same team in this study. The baseline characteristics of the four subgroups were presented and listed in **Table 1**.

**Table 1:** Clinical and pathological characteristics of subjects in this study.

	PTC (n = 80)	PS (n = 86)	PR (n = 9)	HC (n = 80)
Age [y, X ± S]	41.19 ± 8.74	40.31 ± 8.26	41.56 ± 11.92	38.26 ± 6.54
BMI [kg/m^2^, X ± S]	24.12 ± 3.01	23.50 ± 2.14	22.09 ± 1.78	24.56 ± 3.48
Sex [n (%)]				
Female	40 (50.0)	41 (47.7)	8 (88.9)	40 (50.0)
Male	40 (50.0)	45 (52.3)	1 (11.1)	40 (50.0)
Tumor size [n (%)]				**\**
≤ 1	60 (75.0)	57 (66.3)	7 (77.8)	**\**
> 1	20 (25.0)	29 (33.7)	2 (22.2)	**\**
Preop clinical LNM [n (%)]				**\**
Absent	74 (92.5)	77 (89.5)	8 (88.9)	**\**
Present	6 (7.5)	9 (10.5)	1 (11.1)	**\**
Pathological subtype [n (%)]				**\**
Classic	58 (72.5)	67 (77.9)	7 (77.8)	**\**
Follicular variant	8 (10.0)	5 (5.8)	1 (11.1)	**\**
Classic and follicular variant	14 (17.5)	14 (16.3)	1 (11.1)	**\**
Tumor location [n (%)]				**\**
Unifocal	60 (75.0)	58 (67.4)	7 (77.8)	**\**
Multifocal	20 (25.0)	28 (32.6)	2 (22.2)	**\**
Microscopic ETE [n (%)]				**\**
Absent	30 (37.5)	37 (43.0)	3 (33.3)	**\**
Present	50 (62.5)	49 (57.0)	6 (66.7)	**\**
Hashimoto’s thyroiditis [n (%)]				**\**
Absent	62 (77.5)	71 (82.6)	8 (88.9)	**\**
Present	18 (22.5)	15 (17.4)	1 (11.1)	**\**
Postop serum Tg [ng/mL, $\bar{X}$ ± S]	**\**	2.05 ± 4.35	54.41 ± 97.19	**\**

PTC, papillary thyroid cancer; PS, postoperative surveillance; PR, postoperative recurrence; HC, healthy controls; BMI, body mass index; LNM, lymph node metastasis; ETE; Tg, thyroglobulin; Preop, preoperative; Postop, postoperative. Data are presented as mean ± standard deviation or n (%).

The blood samples were collected by percutaneous cubital venipuncture. The whole blood was taken in a centrifuge tube for 2 h at room temperature then centrifuged at 3,000 g for 10 min, and the supernatant was stored at -80°C. Each patient or volunteer donated 3-5 mL serum. The study was approved by the Ethics Committee of Peking Union Medical College Hospital which conformed to the principles outlined in the Declaration of Helsinki and informed written consents from all participants were provided.

### Serum *N*-Glycome Analysis and MS Data Processing

*N*-Glycans were enzymatically released from the serum glycoproteins according to the previously reported protocol (25). Briefly, 5 μl of serum from each specimen were denatured by adding 10 μl of 2% sodium dodecyl sulfonate (SDS) and then incubated at 60°C for 10 min. The procedure of glycan release was conducted by the addition of 10 μl of 2.5 × phosphate-buffered salines (PBS) involving 2% Nonidet P-40 and 1 U PNGase F, followed by incubation at 37°C for 16 h. Sialic acid residues at the non-reducing ends of the glycans were derivatized to end products (α-2,3- linked sialic acids were lactonized and α-2,6-linked sialic acids were ethyl-esterified) in the derivatization process, permitting mass-based differentiation of linkage-specific sialylation. In short, 1 μl of released serum was then added to 20 μl of derivatization reagent containing 250 mM hydroxybenzotriazole (HOBt) and 250 mM 1-ethyl-3-(3-dimethylaminopropyl)-carbodiimide (EDC) in ethanol, followed by incubation at 37°C for 1 h. Subsequently, derivatized glycans were enriched and purified by internally developed cotton-based hydrophilic interaction liquid chromatography solid-phase extraction (HILIC-SPE) micro-tips and eventually eluted into MilliQ (MQ) water (24). Thereafter, 1 μl of the purified glycans were mixed by the matrix (5 mg/ml sDHB in 50% acetonitrile with 1 mM NaOH) on a MALDI target plate and dried by air at room temperature. The Bruker rapifleXtreme MALDI-TOF mass spectrometer was used to measure the derivatized glycans. This equipment was equipped with a Smartbeam-3D laser and controlled by the proprietary software flexControl 4.0 (Bruker Daltonics). The Bruker Peptide Calibration Standard II was used for instrument calibration. The measurements were recorded at the 1,000–5,000 m/z window with 5k laser shots at the frequency of 5,000 Hz in the random walking pattern of 100 shots per raster spot.

MS raw data were processed according to a previously reported workflow (19, 25). The MS glycomics raw data had been deposited in GlycoPOST (ID: GPST000252, https://glycopost.glycosmos.org) (26). Briefly, raw data of all samples was smoothed and baseline-corrected by the software flexAnalysis (Bruker Daltonics). Transformed.XY files were re-calibrated with internally developed MassyTools software (version 0.1.8.1.2) (24). The MS peaks were manually distributed into glycan compositions by the Glyco-Peakfinder tool of GlycoWorkench (27, 28)and the glycan compositions were confirmed by literature (18, 19, 24, 25),. Briefly, peaks (isotope clusters) with a signal-to-noise ratio (S/N) above nine and good isotopic patterns were listed. Next, these peaks (analytes) were annotated with glycan compositions in GlycoWorkbench using the Glyco-Peakfinder tool. A composition list consisting of 131 putative glycan compositions was generated and the peak areas (background-corrected) of each putative glycan composition were targeted extracted using the composition list by MassyTools. During the curation (S/N > 9, ppm error < 20, QC score < 25%, the minimum percentage (>50%) of presence in all spectra of PTC, PS, PR, HC or quality standard samples), 96 out of the 131 glycan compositions passed the quality criteria and were retained for the subsequent quantitatively analysis (**Supplementary Table S1**). Finally, the sum of glycan areas of each spectrum was rescaled to 1 to assess relative intensities. Meanwhile, to explore the glycosylation features including the number of antennae within complex-type *N*-glycans (CA), the level of bisection (B), fucosylation (F), galactosylation (G), and sialylation (S), 91 derived glycan traits were calculated from the 96 directly detected glycan traits based on their structural characteristics. The formulas used for the calculation of the derived glycan traits are given (**Supplementary Table S2**). The calculations were conducted in RStudio.

### Experimental Design and Statistical Analysis

The 255 samples from the cohort (80 PTC, 86 PS, 9 PR, and 80 HC) and 32 quality standard samples (26 serum standards and 6 blanks) were randomly assigned to three 96-well plates and prepared for further analysis. All samples fulfilled the quality requirement. Twenty-six serum standard samples were randomly divided into three 96-well plates to evaluate the data quality of the cohort. For this, the average value, standard deviation (SD), and coefficient of variance (CV) of serum standard samples were calculated for both directly detected glycan and derived glycan traits.

Since the data were non-normally distributed, the nonparametric Mann–Whitney–Wilcoxon test was used to compare direct and derived glycan traits among subgroups (HC vs. PTC, PTC vs. PS, PS vs. PR). Multiple testing correction was used to adjust the significance threshold (P = 0.05/91—the number of derived glycan traits, P = 0.05/96—the number of directly detected glycan traits). The directly detected or derived glycan traits resulting in statistically significant p-values were further analyzed by receiver operating characteristic (ROC) curve test to evaluate their accuracy, sensitivity, and specificity in the prediction of recurrence using GraphPad Prism Software (version 8.4.3). The area under the curve (AUC) of ROC was used to assess the predictive performance of the glycan traits. All statistical analyses were conducted by SPSS statistics (version 25.0).

## Results

### Data Reliability

**Figure 1:**
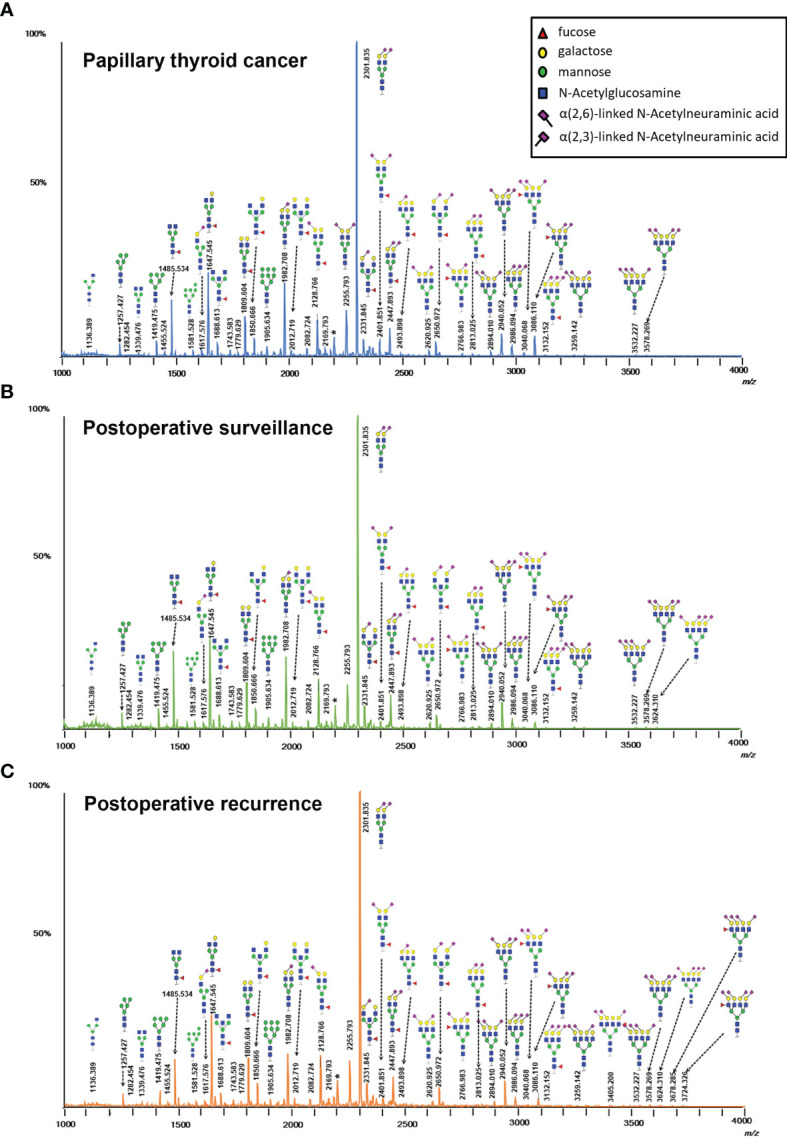
Typical MALDI-TOF-MS spectra of serum protein *N*-glycome for **(A)** papillary thyroid cancer, **(B)** postoperative surveillance, and **(C)** postoperative recurrence. Spectra were recorded in positive-ion reflectron mode on a Bruker rapifleXtreme mass spectrometer. Major *N*-glycan peaks were annotated and assigned to compositions and the presence of structural isomers cannot be excluded. The asterisk (*) are by-products.

The serum *N*-glycomic profiles of patients with PTC, PS, PR, and matched HC volunteers were analyzed by MALDI-TOF MS. Ninety-six of directly detected glycan passed the set-up quality criteria (**Supplementary Table S1**). Multiple directly detected glycan traits were shown differentially expressed between PTC, PS, and PR. Typical annotated MALDI-TOF-MS spectra of serum *N*-glycomes from PTC, PS, and PR were depicted in **Figure 1**, demonstrating differences in peak patterns between the three groups. Ninety-one derived glycan compositions were calculated from 96 directly detected glycan traits based on the similar structural traits of glycans (**Supplementary Table S2**). As noted earlier, derived glycans traits could reflect the biosynthetic process and facilitate the interpretation of biological effects to a certain extent. The average value, SD, and CV from twenty-six serum standard samples were measured together with cohort specimens and also demonstrated method repeatability at the level of directly detected glycan and derived glycan (**Supplementary Table S3**). The MS raw data of directly detected glycan and derived glycan traits of all measured serum samples in the cohort were presented in **Supplementary Table S4**.

### Identification of Serum *N*-Glycome Alteration in PTC, PS, and HC

**Figure 2:**
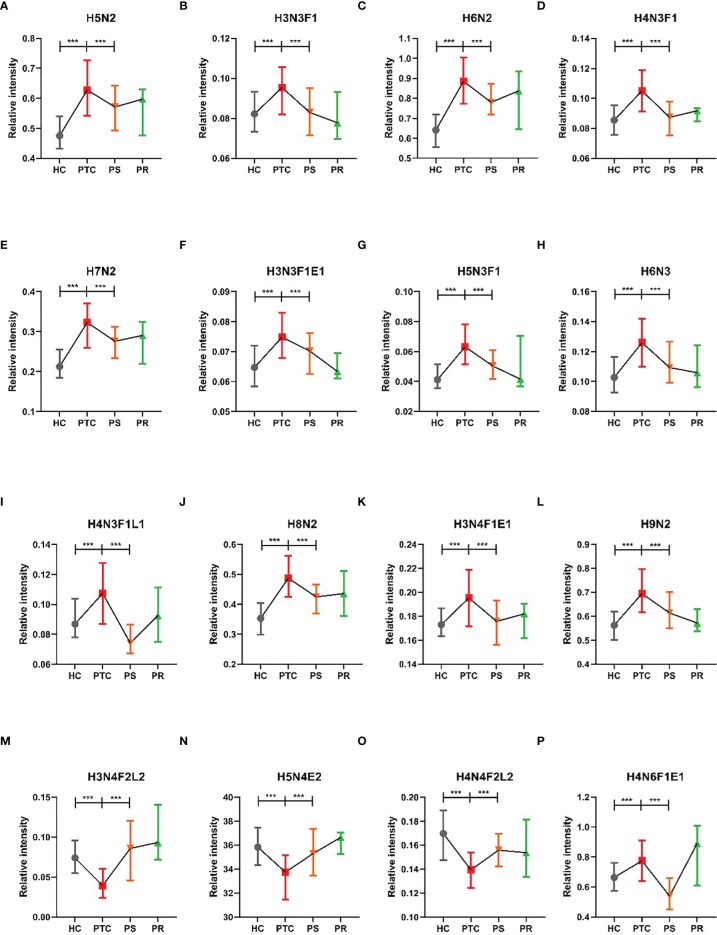
The tendency charts of directly detected glycan traits which response to operation **(A)** H5N2, **(B)** H3N3F1, **(C)** H6N2, **(D)** H4N3F1, **(E)** H7N2, **(F)** H3N3F1E1, **(G)** H5N3F1, **(H)** H6N3, **(I)** H4N3F1L1, **(J)** H8N2, **(K)** H3N4F1E1, **(L)** H9N2, **(M)** H3N4F2L2, **(N)** H5N4E2, **(O)** H4N4F2L2, **(P)** H4N6F1E1, which indicated consecutive changes (HC vs. PTC, PTC vs. PS). Numerical values manifest compositional limitations. See **Supplementary Table S5** for more detailed directly detected glycan traits. The whiskers represent “median with interquartile range”. *** represents *p*-value < 0.001 (after multiple testing correction).

**Figure 3:**
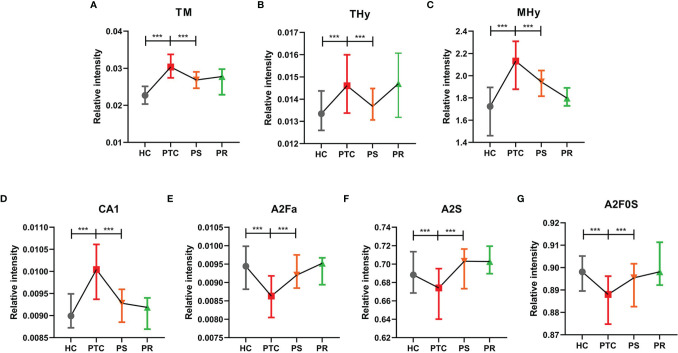
The tendency charts of derived glycan traits **(A)** TM, **(B)** THy, **(C)** MHy, **(D)** CA1, **(E)** A2Fa, **(F)** A2S, **(G)** A2F0S, which indicated consecutive changes (HC vs. PTC, PTC vs. PS). Numerical values manifest compositional limitations. See **Supplementary Table S6** for more detailed derived glycan traits. The whiskers represent “median with interquartile range”. *** represents *p*-value < 0.001 (after multiple testing correction).

To assess the differentially expressed serum *N*-glycans between PTC and HC, 96 directly detected glycan traits and 91 derived glycan traits were compared. A series of glycan traits were found differentially expressed among PTC, PS, and HC. Significant differences of serum *N*-glycans were observed in 31 directly detected glycan traits and 15 derived glycan traits, which could distinguish PTC from health controls (**Supplementary Table S5** and **Supplementary Table S6**). To further explore whether the differentially expressed *N*-glycan traits between PTC and HC respond to initial surgical resection therapy, we compared the PTC group and PS group based on the above differentially expressed glycan traits. Significant differences of *N*-glycans traits between PTC and PS were observed in 16 directly detected glycan traits and 7 derived glycan traits (**Supplementary Table S5** and **Supplementary Table S6**). In the directly detected glycan traits, lower levels were found in PS patients than that in PTC for H5N2, H3N3F1, H6N2, H4N3F1, H7N2, H3N3F1E1, H5N3F1, H6N3, H4N3F1L1, H8N2, H3N4F1E1, H9N2, and H4N6F1E1. In contrast, H3N4F2L2, H5N4E2, and H4N4F2L2 were up-regulated in the PS group compared to the PTC group (**Figure 2**). In the derived glycan traits, TM, THy, MHy, and CA showed significant decreases in PS compared to PTC. In contrast, sialylation and antennary fucosylation (A2Fa, A2S, and A2F0S) were significantly increased in the PS group compared to the PTC group (**Figure 3**). Importantly, these glycans consisting of 16 directly detected glycan traits and 7 derived glycan traits showed the trend toward normal levels (HC group) after therapy (**Figures 2**, **3**).

### Association of Serum *N*-Glycome With PR in PTC

**Figure 4:**
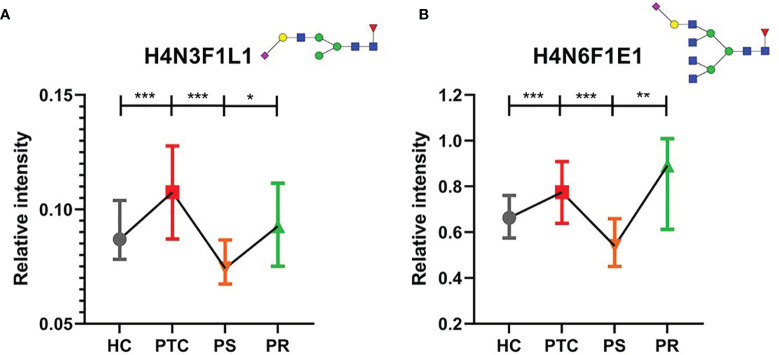
The tendency charts of directly detected glycan traits **(A)** H4N3F1L1, **(B)** H4N6F1E1, which indicated consecutive changes (HC vs. PTC, PTC vs. PS, PS vs. PR). Numerical values manifest compositional limitations. See **Supplementary Table S5** for more detailed directly detected glycan traits. The whiskers represent “median with interquartile range”. *** represents *p*-value < 0.001; ** represents *p*-value < 0.01; * represents *p*-value < 0.05 (after multiple testing correction) Mannose, green circle; galactose, yellow circle; fucose, red triangle; GlcNAc, blue square.

Many directly detected glycan traits responded to therapy. To investigate whether the glycan traits which showed response to therapy have potential in the prediction of recurrence in PTC, we screened these traits by comparing the PR and PS groups. Finally, we found that 2 directly detected glycan traits (H4N3F1L1 and H4N6F1E1) showed potential as biomarkers for monitoring and predicting the recurrence of PTC patients (**Figure 4**, **Supplementary Table S5**). The 2 glycan traits were up-regulated in PTC compared to HC and showed the trend toward normal levels after initial thyroidectomy. Interestingly, the levels of the 2 glycan traits were increased to near the levels of the PTC group again when relapse (**Figure 4**, **Supplementary Table S5**). These results indicated that the 2 directly detected glycan traits (H4N3F1L1 and H4N6F1E1) might serve as novel serum biomarkers for monitoring and predicting the recurrence of PTC following thyroidectomy, as further investigated below.

### Performance of Serum Glycan Traits in Predicting Recurrence of PTC Following Thyroidectomy

**Figure 5:**
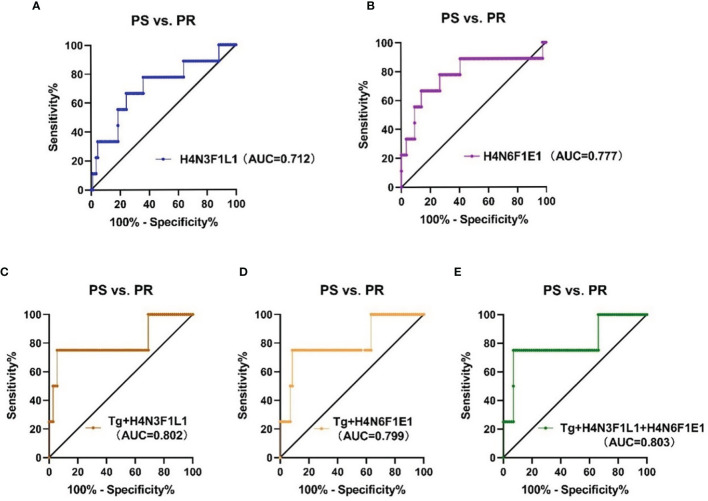
The ROC curve analysis for glycan traits and models (established on glycans and thyroglobulin) in predicting recurrence of PTC **(A, B)** linkage-specific sialylation in predicting recurrence of PTC; **(C)** Tg combined with H4N3F1L1, **(D)** Tg combined with H4N6F1E1, **(E)** Tg combined with H4N3F1L1 and H4N6F1E1. PS, postoperative surveillance; PR, postoperative recurrence; Tg, thyroglobulin.

To assess the accuracy of the 2 directly detected glycan traits in predicting the recurrence of PTC following thyroidectomy, ROC curve analysis was further performed (**Figures 5A, B**). H4N3F1L1 and H4N6F1E1 independently showed moderate accuracy for the predictive power, with AUC values of 0.712 and 0.777, respectively.

Though H4N3F1L1 and H4N6F1E1 showed acceptable performance in the prediction of recurrence in PTC following thyroidectomy, we attempted to explore whether the performance can be further improved when the glycan traits were used together with the clinically existing parameter of postoperative serum Tg level. The performance of the prediction models established based on glycan and serum Tg levels was evaluated by ROC curves (**Figures 5C–E**). The results showed that the AUC value of Tg combined with H4N3F1L1 was 0.802; the AUC value of Tg combined with H4N6F1E1 was 0.799. The performance was further improved when combing Tg with the 2 glycan traits (H4N3F1L1 and H4N6F1E1) with an AUC value of 0.803.

## Discussion

Thyroid cancer is the most common endocrine malignancy, with rising incidence. PTC accounts for more than 85% of all thyroid cancer (29). An in-depth understanding of the pathophysiology and screening of the novel biomarkers for diagnosis and prognosis is quite crucial for PTC. Protein *N*-glycomics profiling is an important posttranslational modification associated with diseases and has the potential as the approach to screen biomarkers. Aberrant glycosylation has been acknowledged as the hallmark of cancer. The glycomic profiling of the thyroid gland is an in-depth area of research, providing new insight into understanding thyroid cancer. To date, limited studies about aberrant glycosylation of thyroid cancer have been reported, which mainly studied aberrant glycosylation changes and focused on tissue, cell, and plasma samples. For example, Kocak et al. analyzed the *N-*glycans profiles of PTC tissues and matched case-control tissues by mass spectrometry and demonstrated that 6 *N*-glycan compositions consisting of 4 sialylated *N-*glycans type and 2 high-mannose types showed the substantial difference between patients and controls (21). In addition, our previous studies found that IgG *N*-glycomic changes (bisecting type neutral *N-*glycans) could distinguish between the benign and malignant thyroid nodules (18). Moreover, we detected aberrant plasma *N-*glycan traits to distinguish benign and malignant thyroid nodules and identify LNM in PTC (19). The published by Kaptan et al. focused on the cell surface of human thyroid cancer cells to determine the alteration of sialylation and fucose expressions. They found that cell surface glycan chains of papillary K1 thyroid carcinoma cells were rich in α-2,3- and α-2,6-linked sialic acid, and α-1,6 fucose residues, indicating that altered cell surface glycosylation in thyroid cancer might be a candidate for novel therapeutic strategies (30). Based on the above and previous studies, altered or aberrant glycosylation is a common phenomenon in thyroid cancer, and it can occur in tumor progression. These dynamic aberrant glycosylation changes also indicated that aberrant glycosylation can serve as biomarkers or treatment targets. However, the existing studies were cross-sectional studies, the serum *N-*glycome of PS or PR patients have never been investigated. In addition, the existing studies were dedicated to finding and reporting the biomarkers of diagnosis, the monitor and prognosis biomarkers have not been reported previously. Finding clinical useful biomarkers to monitor and predict thyroid cancer recurrence the following thyroidectomy is an urgent priority and can help surgeons guide treatment and discuss decisions with patients. Hence, this present work explored the application of MALDI-TOF MS methodology for the assessment of released *N*-glycans from the serum samples of PTC, PS, PR, and HC patients including the linkage-specific sialic acids information to search potential biomarkers for monitoring and predicting the recurrence of PTC following thyroidectomy.

Response to therapy (RTT) system was first proposed by Tuttle et al. in 2010 (31). RTT system was subsequently reported by multiple studies and endorsed by the 2015 American Thyroid Association (ATA) guidelines (32–35). In the present study, we profiled the serum *N*-glycome profiles in PTC, PS, and HC for the first time and found differentially expressed *N-*glycans among three subgroups. At present, significant differences of *N*-glycans traits were observed in 16 directly detected glycan traits and 7 derived glycan traits. These glycan traits changes indicated the response to initial surgical resection therapy in PTC patients, which could be applied to monitor the recurrence after thyroidectomy. Among the above selected directly detected and derived glycan traits, we found that the significant decreases containing α-2,3- and α-2,6-linked sialic acids (H4N3F1L1 and H4N6F1E1) were found in PS compared to PTC. Interestingly, the significant increases in the above differentially expressed glycans were detected in PR. It can be said that PTC patients had significant decreases in the sialylated *N*-glycans including α-2,3- and α-2,6-linked sialic acids following thyroidectomy and significant increases in recurrence. The 2 directly detected glycan traits showed relatively good performance (AUC = 0.712, AUC = 0.777, respectively), indicating serum *N*-glycan patterns have the potential as novel serum biomarkers for predicting the recurrence assisting the existed methods (such as serum Tg, ultrasound, and FNA).

The present study showed the first comprehensive analyses of serum *N-*glycans in PR patients. Several glycosylation compositions were first found to predict recurrence. As for the underlying mechanisms, sialic acids are an important part of monosaccharides associated with cancer-related glycan changes. Aberrant sialylation is involved in the activation and modulation of the immune system and accelerates cancer progression and leads to poor prognosis (36–40). The sialyation further facilitates tumor proliferation and metastasis. Kocak et al. (21) found that all up-regulated *N*-glycans were mainly defined for the sialic acids by analyzing significant changes between PTC tissues and controls, which were consistent with our results. Shah et al. (41) evaluated serum total sialic acid *via* lectins in serum of oral cancer patients and controls and found that the sialylation changes in serum were related to neoplastic transformation and disease progression. Serum sialic acid has been reported in many tumors, which has the potential to be a diagnostic and/or prognostic tumor marker (42–44). Multiple studies have indicated the correlation of the increased level of sialic acids with malignant transformation in the thyroid gland (45–47). We hypothesized that the observed increased linkage-specific sialylation found in PTC and PR in the present study may be implicated in the carcinogenesis or progression of PTC.

Accurate monitoring and prediction of recurrence are particularly critical for postoperative management in PTC patients following thyroidectomy. Waiting for symptomatic recurrence might enhance the treatment morbidity and affect patients’ quality of life. Tg is the dimeric glycoprotein released by normal thyroid follicular tissue and DTC. The regular detection of serum Tg after thyroidectomy indicates either residual thyroid tissue or persistent or recurrent cancer (48). However, serum Tg has a relatively low positive predictive value, false-positive results can affect patients’ mental well-being. Therefore, we combined the 2 glycan traits with serum Tg to explore better prediction approaches. The AUC values of Tg combined with H4N3F1L1 and with H4N6F1E1 were 0.802 and 0.799, respectively. When serum Tg was combined with the 2 glycan traits to predict, it showed improved performance with 0.803 AUC values, suggesting linkage-specific sialylation may have the potential as serum novel biomarkers for predicting the recurrence assisting the existed method (serum Tg). Due to the small sample size of the PR group, the results obtained in the study still need independent validation in future cohorts.

Our study has several limitations. First, the technology of this study doesn’t provide detailed information about serum protein originals of glycans biomarkers. The limitations of current techniques are already known and can be resolved by glycoproteomic analyses. Second, serum samples for patients with PTC and PS groups were not from the same individual, but from the same group. In further studies, the blood draw was collected for each enrolled patient at the time of study enrollment, and at the time of 12 months after surgery to track the aberrant glycosylation changes at different time points. Third, external large validation cohorts and prospective investigations are needed to perform.

## Conclusions

To the best of our knowledge, this is the first study to provide comprehensive evaluations of the serum *N*-glycomic changes in patients with PS or PR including novel linkage-specific sialylation information. Serum glycosylation has proven differences among PTC, PS, and HC, which can help to explore the response of patients to initial surgical resection therapy. Several candidate serum *N*-glycan biomarkers have been determined and shown a high potential for predicting recurrence. Further large-cohort studies are warranted to validate the candidate biomarkers for monitoring and recurrence prediction of PTC.

## Data Availability Statement

The original contributions presented in the study are included in the article/**Supplementary Material**. Further inquiries can be directed to the corresponding authors.

## Ethics Statement

The study was performed according to the guidelines of the Declaration of Helsinki and approved by the Ethics Committee of Peking Union Medical College Hospital(JS-2670). The patients/participants provided their written informed consent to participate in this study.

## Author Contributions

ZC, ZZ, XX, and ZWL conceived and initiated this study. ZC and ZZ performed the experiments and data analysis. RL, MW, and ZPL collected samples and clinical parameters. ZC prepared the figures and tables and wrote the original draft with support from ZZ. All authors contributed to the article and approved the submitted version.

## Funding

This research was supported by the National Nature Science Foundation of China (Nos. 32071436, 82172727, 81572459, and 31901041), Nature Science Foundation of Beijing (Nos. 7202164 and 7222127), and CAMS Innovation Fund for Medical Sciences (CIFMS) (No.2021-I2M-1-002).

## Conflict of Interest

The authors declare that the research was conducted in the absence of any commercial or financial relationships that could be construed as a potential conflict of interest.

## Publisher’s Note

All claims expressed in this article are solely those of the authors and do not necessarily represent those of their affiliated organizations, or those of the publisher, the editors and the reviewers. Any product that may be evaluated in this article, or claim that may be made by its manufacturer, is not guaranteed or endorsed by the publisher.

## References

[1] Siegel RL Miller KD Jemal A. Cancer Statistics, 2019. CA Cancer J Clin (2019) 69(1):7–34. doi: 10.3322/caac.21551 30620402

[2] Jiang LH Yin KX Wen QL Chen C Ge MH Tan Z. Predictive Risk-Scoring Model For Central Lymph Node Metastasis and Predictors of Recurrence in Papillary Thyroid Carcinoma. Sci Rep (2020) 10(1):710. doi: 10.1038/s41598-019-55991-1 31959758 PMC6971227

[3] Ruiz EML Niu T Zerfaoui M Kunnimalaiyaan M Friedlander PL Abdel-Mageed AB. A Novel Gene Panel for Prediction of Lymph-Node Metastasis and Recurrence in Patients With Thyroid Cancer. Surgery (2020) 167(1):73–9. doi: 10.1016/j.surg.2019.06.058 31711617

[4] Ahn D Lee GJ Sohn JH. Recurrence Following Hemithyroidectomy in Patients With Low- and Intermediate-Risk Papillary Thyroid Carcinoma. Br J Surg (2020) 107(6):687–94. doi: 10.1002/bjs.11430 32026467

[5] Chereau N Oyekunle TO Zambeli-Ljepovic A Kazaure HS Roman SA Menegaux F. Predicting Recurrence of Papillary Thyroid Cancer Using the Eighth Edition of the AJCC/UICC Staging System. Br J Surg (2019) 106(7):889–97. doi: 10.1002/bjs.11145 PMC6825520 31012500

[6] Hong CM Lee WK Jeong SY Lee SW Ahn BC Lee J. Superiority of Delayed Risk Stratification in Differentiated Thyroid Cancer After Total Thyroidectomy and Radioactive Iodine Ablation. Nucl Med Commun (2014) 35(11):1119–26. doi: 10.1097/MNM.0000000000000183 25144561

[7] Evans C Tennant S Perros P. Serum Thyroglobulin in the Monitoring of Differentiated Thyroid Cancer. Scand J Clin Lab Invest Suppl (2016) 245:S119–23. doi: 10.1080/00365513.2016.1210339 27542000

[8] Li F Pan D He Y Wu Y Peng J Li J. Using Ultrasound Features and Radiomics Analysis to Predict Lymph Node Metastasis in Patients With Thyroid Cancer. BMC Surg (2020) 20(1):315. doi: 10.1186/s12893-020-00974-7 33276765 PMC7716434

[9] Phan HT Jager PL van der Wal JE Sluiter WJ Plukker JT Dierckx RA. The Follow-Up of Patients With Differentiated Thyroid Cancer and Undetectable Thyroglobulin (Tg) and Tg Antibodies During Ablation. Eur J Endocrinol (2008) 158(1):77–83. doi: 10.1530/EJE-07-0399 18166820

[10] Zahra HO Omran GA Gewely AG Eldehn AF Abdo W Elmahallawy EK. Prognostic Value of Serum Thyroglobulin and Anti-Thyroglobulin Antibody in Thyroid Carcinoma Patients Following Thyroidectomy. Diagn (Basel) (2021) 11(11):2080. doi: 10.3390/diagnostics11112080 PMC8622548 34829426

[11] Knappe L Giovanella L. Life After Thyroid Cancer: The Role of Thyroglobulin and Thyroglobulin Antibodies for Postoperative Follow-Up. Expert Rev Endocrinol Metab (2021) 16(6):273–9. doi: 10.1080/17446651.2021.1993060 34693849

[12] Moremen KW Tiemeyer M Nairn AV. Vertebrate Protein Glycosylation: Diversity, Synthesis and Function. Nat Rev Mol Cell Biol (2012) 13(7):448–62. doi: 10.1038/nrm3383 PMC3934011 22722607

[13] Hart GW Copeland RJ. Glycomics Hits the Big Time. Cell (2010) 143(5):672–6. doi: 10.1016/j.cell.2010.11.008 PMC3008369 21111227

[14] Scott DA Drake RR. Glycosylation and its Implications in Breast Cancer. Expert Rev Proteomics (2019) 16(8):665–80. doi: 10.1080/14789450.2019.1645604 PMC6702063 31314995

[15] Hakomori S. Tumor-Associated Carbohydrate Antigens Defining Tumor Malignancy: Basis for Development of Anti-Cancer Vaccines. Adv Exp Med Biol (2001) 491:369–402. doi: 10.1007/978-1-4615-1267-7_24 14533809

[16] Park HM Hwang MP Kim YW Kim KJ Jin JM Kim YH. Mass Spectrometry-Based *N*-Linked Glycomic Profiling as a Means for Tracking Pancreatic Cancer Metastasis. Carbohydr Res (2015) 413:5–11. doi: 10.1016/j.carres.2015.04.019 26057990

[17] Yue T Goldstein IJ Hollingsworth MA Kaul K Brand RE Haab BB. The Prevalence and Nature of Glycan Alterations on Specific Proteins in Pancreatic Cancer Patients Revealed Using Antibody-Lectin Sandwich Arrays. Mol Cell Proteomics (2009) 8(7):1697–707. doi: 10.1074/mcp.M900135-MCP200 PMC2709194 19377061

[18] Zhang Z Wu J Liu P Kang L Xu X. Diagnostic Potential of Plasma IgG N-Glycans in Discriminating Thyroid Cancer From Benign Thyroid Nodules and Healthy Controls. Front Oncol (2021) 11:658223. doi: 10.3389/fonc.2021.658223 34476207 PMC8406750

[19] Zhang Z Reiding KR Wu J Li Z Xu X. Distinguishing Benign and Malignant Thyroid Nodules and Identifying Lymph Node Metastasis in Papillary Thyroid Cancer by Plasma *N*-Glycomics. Front Endocrinol (Lausanne) (2021) 12:692910. doi: 10.3389/fendo.2021.692910 34248851 PMC8267918

[20] Chen G Wang Y Qiu L Qin X Liu H Wang X. Human IgG Fc-Glycosylation Profiling Reveals Associations With Age, Sex, Female Sex Hormones and Thyroid Cancer. J Proteomics (2012) 75(10):2824–34. doi: 10.1016/j.jprot.2012.02.001 22365975

[21] Kocak OF Kayili HM Albayrak M Yaman ME Kadioglu Y Salih B. N-Glycan Profiling of Papillary Thyroid Carcinoma Tissues by MALDI-TOF-MS. Anal Biochem (2019) 584:113389. doi: 10.1016/j.ab.2019.113389 31400301

[22] Miyoshi E Ito Y Miyoshi Y. Involvement of Aberrant Glycosylation in Thyroid Cancer. J Oncol (2010) 2010:816595. doi: 10.1155/2010/816595 20652009 PMC2906155

[23] Bastian K Scott E Elliott DJ Munkley J. FUT8 Alpha-(1,6)-Fucosyltransferase in Cancer. Int J Mol Sci (2021) 22(1):455. doi: 10.3390/ijms22010455 PMC7795606 33466384

[24] Jansen BC Reiding KR Bondt A Hipgrave Ederveen AL Palmblad M Falck D. MassyTools: A High-Throughput Targeted Data Processing Tool for Relative Quantitation and Quality Control Developed for Glycomic and Glycoproteomic MALDI-Ms. J Proteome Res (2015) 14(12):5088–98. doi: 10.1021/acs.jproteome.5b00658 26565759

[25] Reiding KR Blank D Kuijper DM Deelder AM Wuhrer M. High-Throughput Profiling of Protein *N*-Glycosylation by MALDI-TOF-MS Employing Linkage-Specific Sialic Acid Esterification. Anal Chem (2014) 86(12):5784–93. doi: 10.1021/ac500335t 24831253

[26] Watanabe Y Aoki-Kinoshita KF Ishihama Y Okuda S. GlycoPOST Realizes FAIR Principles for Glycomics Mass Spectrometry Data. Nucleic Acids Res (2021) 49(D1):D1523–8. doi: 10.1093/nar/gkaa1012 PMC7778884 33174597

[27] Ceroni A Maass K Geyer H Geyer R Dell A Haslam SM. GlycoWorkbench: A Tool for the Computer-Assisted Annotation of Mass Spectra of Glycans. J Proteome Res (2008) 7(4):1650–9. doi: 10.1021/pr7008252 18311910

[28] Maass K Ranzinger R Geyer H von der Lieth CW Geyer R. “Glyco-Peakfinder”–*De Novo* Composition Analysis of Glycoconjugates. Proteomics (2007) 7(24):4435–44. doi: 10.1002/pmic.200700253 18072204

[29] Davies L Welch HG. Current Thyroid Cancer Trends in the United States. JAMA Otolaryngol Head Neck Surg (2014) 140(4):317–22. doi: 10.1001/jamaoto.2014.1 24557566

[30] Kaptan E Sancar-Bas S Sancakli A Bektas S Bolkent S. The Effect of Plant Lectins on the Survival and Malignant Behaviors of Thyroid Cancer Cells. J Cell Biochem (2018) 119(7):6274–87. doi: 10.1002/jcb.26875 29663501

[31] Tuttle RM Tala H Shah J Leboeuf R Ghossein R Gonen M. Estimating Risk of Recurrence in Differentiated Thyroid Cancer After Total Thyroidectomy and Radioactive Iodine Remnant Ablation: Using Response to Therapy Variables to Modify the Initial Risk Estimates Predicted by the New American Thyroid Association Staging System. Thyroid (2010) 20(12):1341–9. doi: 10.1089/thy.2010.0178 PMC4845674 21034228

[32] Pitoia F Jerkovich F Urciuoli C Schmidt A Abelleira E Bueno F. Implementing the Modified 2009 American Thyroid Association Risk Stratification System in Thyroid Cancer Patients With Low and Intermediate Risk of Recurrence. Thyroid (2015) 25(11):1235–42. doi: 10.1089/thy.2015.0121 26132983

[33] Jeon MJ Kim WG Park WR Han JM Kim TY Song DE. Modified Dynamic Risk Stratification for Predicting Recurrence Using the Response to Initial Therapy in Patients With Differentiated Thyroid Carcinoma. Eur J Endocrinol (2014) 170(1):23–30. doi: 10.1530/EJE-13-0524 24088549

[34] Castagna MG Maino F Cipri C Belardini V Theodoropoulou A Cevenini G. Delayed Risk Stratification, to Include the Response to Initial Treatment (Surgery and Radioiodine Ablation), has Better Outcome Predictivity in Differentiated Thyroid Cancer Patients. Eur J Endocrinol (2011) 165(3):441–6. doi: 10.1530/EJE-11-0466 21750043

[35] Ritter A Mizrachi A Bachar G Vainer I Shimon I Hirsch D. Detecting Recurrence Following Lobectomy for Thyroid Cancer: Role of Thyroglobulin and Thyroglobulin Antibodies. J Clin Endocrinol Metab (2020) 105(6):dgaa152. doi: 10.1210/clinem/dgaa152 32219303

[36] Karmakar J Mandal C. Interplay Between Sialic Acids, Siglec-E, and Neu1 Regulates MyD88- and TRIF-Dependent Pathways for TLR4-Activation During Leishmania Donovani Infection. Front Immunol (2021) 12:626110. doi: 10.3389/fimmu.2021.626110 33763070 PMC7982817

[37] McCurdy TR Bhakta V Eltringham-Smith LJ Gataiance S Fox-Robichaud AE Sheffield WP. *In Vivo* Clearance of Alpha-1 Acid Glycoprotein is Influenced by the Extent of Its *N*-Linked Glycosylation and by its Interaction With the Vessel Wall. J BioMed Biotechnol (2012) 2012:292730. doi: 10.1155/2012/292730 22545002 PMC3321579

[38] Varki A Gagneux P. Multifarious Roles of Sialic Acids in Immunity. Ann N Y Acad Sci (2012) 1253:16–36. doi: 10.1111/j.1749-6632.2012.06517.x 22524423 PMC3357316

[39] Zhou X Yang G Guan F. Biological Functions and Analytical Strategies of Sialic Acids in Tumor. Cells. (2020) 9(2):273. doi: 10.3390/cells9020273 PMC7072699 31979120

[40] Zhang Z Wuhrer M Holst S. Serum Sialylation Changes in Cancer. Glycoconj J (2018) 35(2):139–60. doi: 10.1007/s10719-018-9820-0 PMC5916985 29680984

[41] Shah MH Telang SD Shah PM Patel PS. Tissue and Serum Alpha 2-3- and Alpha 2-6-Linkage Specific Sialylation Changes in Oral Carcinogenesis. Glycoconj J (2008) 25(3):279–90. doi: 10.1007/s10719-007-9086-4 18158621

[42] Mensah SA Harding IC Zhang M Jaeggli MP Torchilin VP Niedre MJ. Metastatic Cancer Cell Attachment to Endothelium is Promoted by Endothelial Glycocalyx Sialic Acid Degradation. AIChE J (2019) 65(8):e16634. doi: 10.1002/aic.16634 31367063 PMC6668365

[43] Ding J Zhao D Hu Y Liu M Liao X Zhao B. Terminating the Renewal of Tumor-Associated Macrophages: A Sialic Acid-Based Targeted Delivery Strategy for Cancer Immunotherapy. Int J Pharm (2019) 571:118706. doi: 10.1016/j.ijpharm.2019.118706 31593811

[44] Patel PS Rawal GN Balar DB. Importance of Serum Sialic Acid and Lactate Dehydrogenase in Diagnosis and Treatment Monitoring of Cervical Cancer Patients. Gynecol Oncol (1993) 50(3):294–9. doi: 10.1006/gyno.1993.1214 8406190

[45] Takeyama H Kyoda S Okamoto T Manome Y Watanabe M Kinoshita S. The Expression of Sialic Fibronectin Correlates With Lymph Node Metastasis of Thyroid Malignant Neoplasmas. Anticancer Res (2011) 31(4):1395–8. doi: 10.1007/s13277-010-0136-3 21508391

[46] Ghosh S. Sialic Acids: Biomarkers in Endocrinal Cancers. Glycoconj J (2015) 32(3-4):79–85. doi: 10.1007/s10719-015-9577-7 25777812

[47] Vierbuchen M Schroder S Larena A Uhlenbruck G Fischer R. Native and Sialic Acid Masked Lewis(a) Antigen Reactivity in Medullary Thyroid Carcinoma. Distinct Tumour-Associated and Prognostic Relevant Antigens. Virchows Arch (1994) 424(2):205–11. doi: 10.1007/BF00193501 8180782

[48] Gray JL Singh G Uttley L Balasubramanian SP. Routine Thyroglobulin, Neck Ultrasound and Physical Examination in the Routine Follow Up of Patients With Differentiated Thyroid Cancer-Where Is the Evidence? Endocrine (2018) 62(1):26–33. doi: 10.1007/s12020-018-1720-3 30128957 PMC6153587

